# Acupuncture for knee osteoarthritis with sensitized acupoints: results from a pilot, feasibility randomized controlled trial

**DOI:** 10.1186/s40814-020-00687-x

**Published:** 2020-09-28

**Authors:** Pengli Jia, Jiali Liu, Ling Li, Yanan Luo, Ying Li, Ling Zhao, Fanrong Liang, Zhibin Liu, Kang Zou, Li Tang, Xin Sun

**Affiliations:** 1 Chinese Evidence-based Medicine Center and Cochrane China Center, West China Hospital, Sichuan University, 37 Guo Xue Xiang, Chengdu, 610041 Sichuan China; 2 Acupuncture and Tuina School, Chengdu University of Traditional Chinese Medicine, Chengdu, 610000 Sichuan China

**Keywords:** Acupuncture, Knee osteoarthritis, Sensitized acupoints

## Abstract

**Background:**

Although previous clinical studies suggest possible benefits of acupuncture for knee osteoarthritis (KOA), the value of acupuncture at sensitized points is uncertain. We aimed to preliminarily assess the feasibility of performing a definitive randomized controlled trial to explore the effectiveness of acupuncture for KOA with highly sensitized acupoints.

**Methods:**

In this randomized, single-blind, parallel, pilot trial, 36 participants with KOA were randomly assigned to receive acupuncture at highly sensitized acupoints (high-sensitization group) or at low/non-sensitized points (low/non-sensitization group) by a computer-generated random sequence. Both groups received three treatment sessions per week for four consecutive weeks (12 sessions in total). Assessments were performed at screening and at 4, 8, 12, and 16 weeks after randomization. Primary feasibility outcomes were patient recruitment, retention rate, and adherence to group treatment. Secondary outcomes included the change of Western Ontario and McMaster Universities Osteoarthritis Index (WOMAC) total score from baseline to 16 weeks, the change of Short Form (SF)-12 health survey score, and safety outcomes.

**Results:**

Patient recruitment of 36 patients took 2 months, achieving the recruitment target. Retention rates were similar between the treatment groups, 14 (77.8%) patients in the high-sensitization group completed the 16-week follow-up and compared to 14 (77.8%) patients in low/non-sensitization group, but the result was lower than expected. All patients received at least ten treatment sessions in total. The WOMAC total score and the pain, stiffness, and physical function score in the high-sensitization group were lower or very close to those in the control group at each assessment point. Similar results were observed on quality of life. No adverse events occurred.

**Conclusion:**

This trial has presented preliminary data on the feasibility of conducting a large trial to test the effectiveness of acupuncture at sensitized points in KOA patients.

**Trial registration:**

ClinicalTrials.gov, NCT03008668. Registered on 26 December 2016—retrospectively registered.

## Background

Osteoarthritis is the leading cause of disability among older adults [1], and the most frequently affected location is the knee [2]. Knee osteoarthritis (KOA) is characterized with limitations in joint movement, worsening pain, and reduction in quality of life [3]. The prevalence, disability, and associated costs of KOA will continue growing over the next 25 years due to population aging [1]. KOA is often managed with medical or surgical treatments [4]. However, long-term use of drugs is associated with significant adverse effects and knee surgery is costly with limited effects [5–7]. A large number of patients with KOA have increasingly used complementary and alternative treatments, particularly acupuncture [8]. For instance, almost 30~40% of general practices have offered complementary therapies for patients with KOA in the UK, and acupuncture is one of the most popular choices [9].

Although previous clinical studies suggested possible benefits of acupuncture for KOA, the value of acupuncture for KOA is yet to be established [10, 11]. Most importantly, the majority of published trials suffered from methodological defects [12–14], such as lack of explicit and consistent disease definition [12], failure to conceal treatment allocation, absence of standards for practicing acupuncture [13], inadequate blinding, small sample size, short follow-up, and poor measurement of outcomes [14]. As a result, the findings were not reliable, and high-quality randomized controlled trials (RCTs) are warranted.

Acupuncture is a complex intervention in nature [15]; its effects are subject to many factors. One important factor is the selection of acupuncture manipulating points (acupoints) [16]. Although acupoints are usually fixed along meridians, evidence suggested that manipulating fixed acupoints may not achieve optimal therapeutic effects [17]. According to the theory of traditional Chinese medicine (TCM), disease condition has an underlying connection with acupoints (i.e., traditional acupoints and tender points (ashi points)), which is manifested on the surface of the human body [18]. When the body is ill, the acupoints associated with that particular condition become sensitized [19], and the sensitized points are characterized with changes of feeling around their skin surface (e.g., red, swollen, heat, pain) [20].

One potentially important implication of sensitized points is that the human body may exert significant response to a small external stimulation (i.e., acupuncture on such points). This means that a sensitized point is not only a pathological phenomenon reflecting the presence of disease state but may represent an optimal target for acupuncture [17]. This phenomenon is often so-called small stimulation inducing large response [19], although the underlying biological rational remains unclear. Various types of sensitization may be presented, such as pain, heat, force, and light [19, 21–24]. One most common and measurable type is pain. Pain-sensitized points are areas that would respond as a result of stimulation of minimum force applied on the skin by external pressure [24]. These areas may be either an acupoint or tender points.

Previous empirical evidence from observational studies have demonstrated that KOA patients are often presented with sensitized points, particularly responsive to pain [25–27]. One may hypothesize that if these pain-sensitized points are identified, the therapeutic effect may be improved for KOA patients receiving acupuncture. However, up to now, studies of pain-sensitized acupoints are typically observational or even theoretical. Little evidence is available to support the hypothesis.

Therefore, we have proposed to conduct a well-designed and conducted and adequately powered RCT to test whether acupuncture at higher sensitized points, compared to low/non-sensitized points, are associated with improved clinical outcomes (e.g., improvement of pain, joint function, and quality of life) in KOA patients. The current pilot study aimed to assess the feasibility of performing a large-scale RCT, including patient recruitment, retention, and adherence to treatment.

## Methods

### Study design and setting

A randomized, single-blind, two-arm, parallel, pilot trial was conducted at the department of acupuncture and moxibustion of the Affiliated Hospital of Chengdu University of Traditional Chinese Medicine in Sichuan province, China. The department has 20 experienced acupuncturists and has treated at least 2000 patients with KOA in the past 3 years.

Eligible and consented patients with KOA were randomly assigned (1:1) to receive acupuncture intervention at high or low/non-sensitized points for 4 weeks. Participants were assessed at baseline and 4, 8, 12, and 16 weeks post-randomization. The primary purpose was to determine the feasibility of conducting a large multi-center RCT of acupuncture treatment for KOA with sensitized points. The study protocol was approved by the Bioethics Committee of West China Hospital, Sichuan University. The trial is registered at ClinicalTrials.gov (NCT03008668).

### Participants

Eligible patients were those aged 40 years or older who were diagnosed with KOA and consented to participate in the trial. The diagnosis of KOA was based on the following criteria [28]: (1) refractory knee pain for most days in the past month; (2) joint space narrowing, sclerosis, or cystic change in the subchondral bone (as indicated by X-ray); (3) laboratory examinations of arthritis: clear and viscous synovial fluid (≥ 2 times) and white blood cell count < 2 × 10^9^/L; (4) age 40 years or older; (5) morning stiffness lasting for less than 30 min; and (6) bone sound exists when joints were taking flexion and/or extension. If a patient meets one of the following combinations of items (1 and 2), (1, 3, and 5), or (1, 4, 5, and 6), a diagnosis of KOA was confirmed.

Patients were excluded if they met any of the following: (1) diagnosed with conditions leading to skeletal disorders, such as tuberculosis, tumors, or rheumatism of the knee joint and rheumatoid arthritis; (2) present with sprain or trauma in the lower limb; (3) unable to walk properly due to foot deformity or pain; (4) present with mental disorders; (5) present with comorbidities including severe cardiovascular disease, liver or kidney impairment, immunodeficiency, diabetes mellitus, or blood disorder; (6) females who are pregnant or lactating; (7) were using physiotherapy treatments for osteoarthritis knee pain; (8) had used intra-articular injection of glucocorticoid or viscosupplementation in the past 6 months; (9) received knee-replacement surgery; and (10) were participating or had participated in the other clinical trials.

### Recruitment and screening

Participating physicians and research staff, thorough physical examination and clinical tests, screened visiting patients for potential eligibility. Eligible patients were informed of the study aims and nature, both verbally and in written form via an information sheet. Participation in the study depended entirely on the patient. Patients may refuse to participate or withdraw from the study at any time during the study period. Informed consent was obtained from all eligible patients. Confidentiality of the information provided by participants and their right to withdraw without prejudice were guaranteed throughout the pilot trial duration. We documented the log of all potential participants, and the main reasons for exclusion, declines, or withdrawals.

### Randomization

Eligible patients consented to participate were randomly assigned to receive acupuncture treatment at highly sensitized acupoints (high-sensitization group) or at low/no-sensitized points (low/non-sensitization group, control) by a computer-generated random sequence using a block length of 4, with the allocation ratio of 1:1. The random allocation sequence was generated by an independent statistician at the main research unit and handed over to the research assistant at the hospital using sequentially numbered, opaque, sealed envelopes. The study coordinator double-checked that the informed consent form had been obtained from each participant prior to assignment.

### Interventions

#### Identification of points for measuring the level of sensitization

We identified 12 candidate acupoints for treating KOA according to previous studies [29, 30] and expert consensus. They are Lingquan (GB34), Dubi (ST35), Nei xiyan (EX-LE4), Zu sanli (ST36), Xue Hai (SP10), Liang Qiu (ST34), Yin Lingquan (SP9), He Ding (EX-LE2), Wei Zhong (BL40), YINGU (KI10) Weiyang (BL39), Xiguan (LR7), and Ququan (LR8).

#### Selection of tender points on the knee regions

**Fig. 1:**
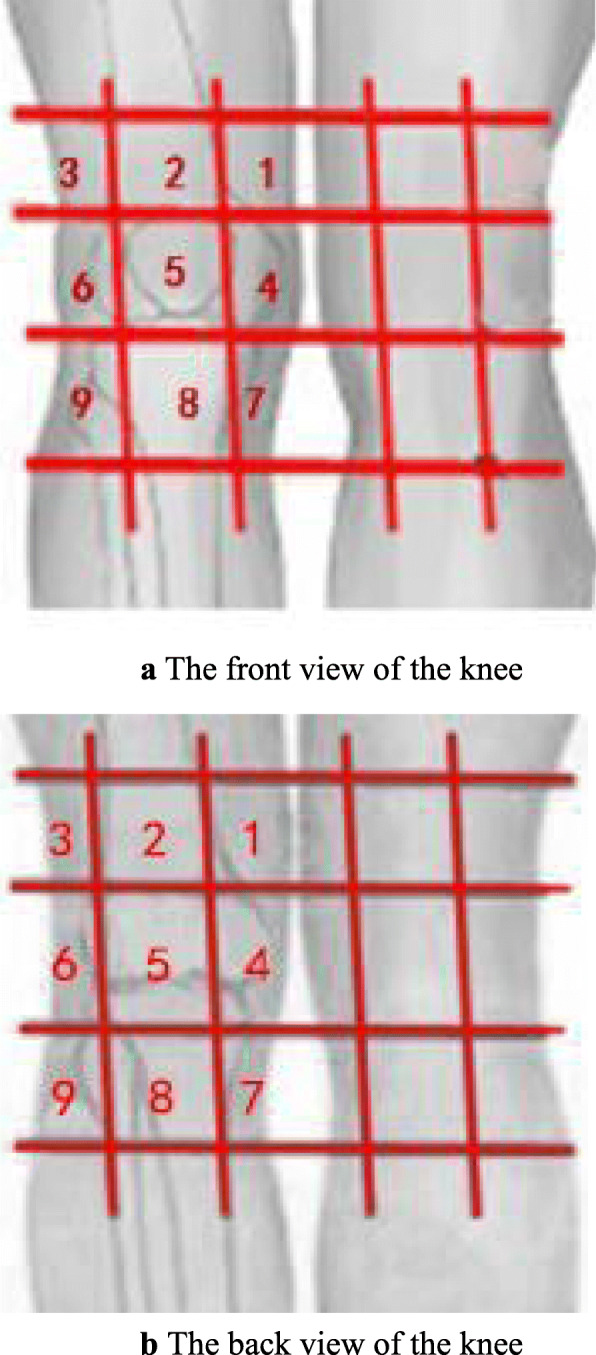
**a** The front view of the knee. **b** The back view of the knee

The front and back of knee joint regions were divided into 12 testing areas. The partition method was as follows: patients were first asked to extend lower limbs; the level of the 7.62 cm above the patella was used as the upper boundary, and the level of ZUSANLI (ST36) as the lower boundary. The central zone was marked by four lines around the knee joint, which were extended through the area. At the front view of the knee, these four lines surround the patella (Fig. 1a); the vertical lines of the posterior knee are at $$ \raisebox{1ex}{$1$}\!\left/ \!\raisebox{-1ex}{$3$}\right. $$ and $$ \raisebox{1ex}{$2$}\!\left/ \!\raisebox{-1ex}{$3$}\right. $$ of the popliteal area (Fig. 1b). Tender points in the testing areas were identified when the participant reports the sensation of pain, soreness, distention, or sometimes comfort while the acupuncturist pressed vertically downward about 6~10 millimeter (mm) at an even speed with the thumb.

#### Measurements of sensitization of identified points

The Von Frey detector (2390-type, IITC Inc. Life Science, USA) was used to detect the pressure-pain threshold of the 12 traditional acupoints and tender points identified in the 12 test zones. The trained operator held the detector, and the probe tip was touched vertically downward on the tender points. Once the patient felt pain, the probe was immediately removed and the value on the detector was recorded. Each point was tested three times within 5 min. The average value of three measurements was recorded as the pain threshold of the tested point.

#### Ranking of sensitization of the identified points

The acupoints and identified tender points were ranked by the pain threshold. The five points with the lowest pain threshold were marked as highly sensitized points, and the five with the highest pain threshold as low or non-sensitized points.

#### Acupuncture at highly sensitized points (intervention group)

Patients in the intervention group received acupuncture treatment at the five highly sensitized points/acupoints. Sterile, disposable needles (Hwato Needles, Sino-foreign Joint Venture Suzhou Hwato Medical Instruments Co., China) with a length of 40 mm and a diameter of 0.30 mm were used. Patients were positioned on the bed, supported by two pillows under the knees. They were instructed not to move during the stimulation period, while staying in a comfortable position. After the local area was disinfected, a trained acupuncturist inserted the needles at a depth of 25 to 40 mm vertically. The stimulation was performed with lifting and thrusting in combination with twirling and rotating for producing the sensation known as “Deqi” [31]. The needles placed in the acupoints were stimulated manually every 15 min and removed after 30 min. Patients received three treatment sessions per week (every other day) for four consecutive weeks (12 sessions in total).

#### Acupuncture at low/non-sensitized points (control group)

Patients in the control group received acupuncture treatment at the five low/non-sensitized points. All other treatment settings were the same as in the intervention group.

Acupuncture treatment was performed by certified acupuncturists at the research hospital. All the participants were discouraged from receiving any other treatments for KOA. However, nonsteroidal anti-inflammatory drugs (NSAIDs) were allowed if patients had intolerable pain and the outcome assessment was not scheduled in the next 48 hours. All such treatments (including drug name, dosage, and duration of treatment) that patients received were documented during the study.

### Follow-up

The pain, stiffness, and physical function of knee joint, quality of life of all the participants, and the safety of treatment were measured at 4, 8, 12, and 16 weeks after randomization. We used the following approaches to maximize the retention of participants: text messages and phone calls to remind participants about upcoming appointments and flexibility in making appointment to suit participants’ schedule. If the appointments were overdue, we made phone calls to patients to request follow-up visits.

### Outcomes

#### Primary outcome

Feasibility outcomes were patient recruitment, retention rate, and adherence to treatment. Patient recruitment was defined as months to recruit 36 patients. Retention rate was defined as the percentage of patients enrolled at baseline, who completed all follow-up measurements. Adherence to treatment was assessed using attendance at the treatment sessions. Successful pilot targets were defined as recruitment of 36 participants over 2 months, 80% retention rate, and at least 10 treatment sessions attended.

#### Secondary outcomes

The secondary study outcomes were the change of Western Ontario and McMaster Universities Osteoarthritis Index (WOMAC) total score from baseline to 16 weeks, the change of Short Form (SF)-12 health survey score from baseline to 16 weeks, and the safety outcomes.

The WOMAC, a reliable instrument, has been translated and validated in different languages and used widely in various clinical trials for KOA [32]. The Chinese version of WOMAC consists of 24 items, which assess pain (5 items), stiffness (2 items), and physical function (17 items) [33]. Each item is graded on a visual analog scale (VAS) ranging from 0 to 10, with higher scores representing more pain, stiffness, and poorer physical function [34].

The SF-12, a shorter version of the SF-36, has been used to measure health-related quality of life for patients with KOA [35]. It includes 12 items: 2 items on physical functioning (PT), 2 items on role limitations because of physical problems (RP), 1 item on bodily pain (BP), 1 item on general health perceptions (GH), 1 item on vitality (VT), 1 item on social functioning (SF), 2 items on role limitations because of emotional problems (RE), and 2 items on general mental health (MH, psychological distress and psychological well-being). The validated Chinese version of SF-12 was used in the present trial [36].

Adverse events (AEs), such as bleeding, nausea/vomiting, fainting, bruising at needle sites, feeling hot/burning, or transient pain at needle sites, were collected through case report forms at 4 weeks after the acupuncture treatment and followed at 8, 12, and 16 weeks. We defined serious adverse events (SAEs) as death, hospitalization, significant disability or incapacity, any life-threatening situations, or any other medically significant events that were potentially related to the trial procedures or acupuncture treatments. The AEs or SAEs were identified by the certified treating acupuncturists.

Both WOMAC and SF-12 were self-administered, with an outcome assessor available for explanation if required. Trained nurses, blinded to treatment allocation, performed outcome assessment in a separate space at the outpatient department of the research site. The outcome assessors were trained on conducting interviews and performing measurements before the pilot study began and followed a standard protocol.

### Blinding

Patients were blinded to treatment assignment. Acupuncturists who selected acupuncture points, measured the pressure-pain threshold, and conducted acupuncture treatment were aware of the treatment allocation. The outcome assessors and the statistician were fully blinded to randomization and were not involved in the treatment process.

### Sample size

The purpose of this study was to evaluate the study feasibility and support the development of a future definitive RCT. Based on the consensus of the methodologists, statisticians, and acupuncturists, we decided that a sample size of 36 was sufficient to examine the study feasibility and collect preliminary outcome data.

### Statistical analysis

Descriptive statistics were used for primary feasibility outcomes (patient recruitment, retention rate, and adherence to treatment) and baseline characteristics. We reported number and percentage for categorical variables, and mean and standard deviation (SD) (or median and interquartile range [IQR]) for continuous variables. For the feasibility outcome about group treatment, we reported mean and 95% confidence intervals. For baseline characteristics, we also did group comparisons using the Wilcoxon rank sum test for continuous data and chi-square or Fisher’s exact test for categorical variables. All statistical tests were two-sided, and the level of significance was set at 0.05.

To explore potential effectiveness, we used repeated measures analysis of variance (ANOVA) models to assess changes in pain and quality of life over time (time main effect) and to investigate whether these changes over time differed between the two groups (interaction of group by time). We used the square root transformation to normalize the outcome variables, and these transformed outcome variables were included in the repeated measures ANOVA. The analyses were performed on the per-protocol (PP) population, which included all eligible patients who completed the treatment originally allocated. Since most participants were lost to follow-up at the early stage for unknown reasons, intention-to treatment analysis was not used. The data was analyzed using SPSS version 21.0 (IBM, Armonk, NY, USA). As this is a pilot study, our results were considered exploratory.

## Results

### Enrollment and participant flow

A total of 56 participants were screened for eligibility between October 2016 and December 2016. Of these, 20 were excluded (14 did not meet the inclusion criteria and 6 declined to participate). In total, 36 patients were included (acceptance rate of 64.3%) and randomly assigned to receive acupuncture on high-sensitized (*n* = 18) or low/non-sensitized points (*n* = 18).

**Fig. 2:**
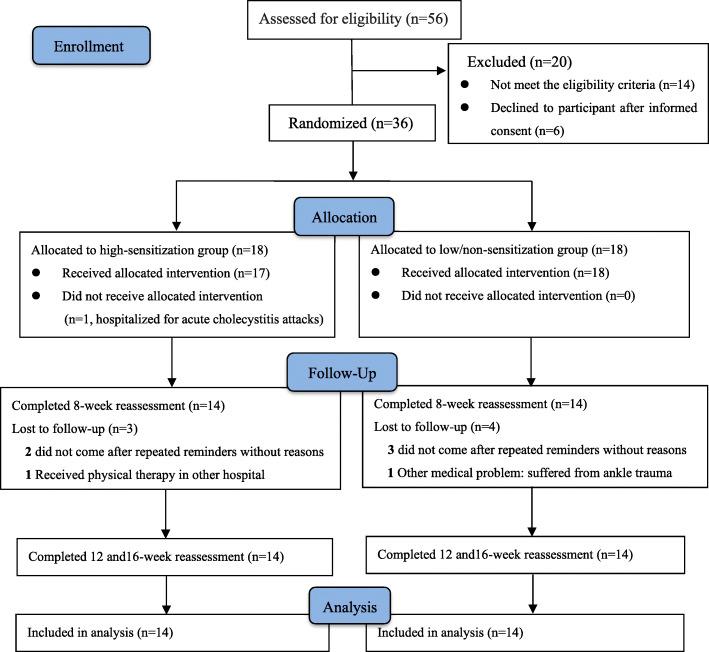
Flow diagram showing patient enrolment and follow-up

At 16 weeks, 28 (77.8%) completed the study and eight dropped out. Following randomization, one patient in the high-sensitization group withdrew due to acute cholecystitis and did not receive treatment. Five were lost to follow-up between 4 and 8 weeks for unknown reasons (two in intervention and three in the control group). In addition, one patient in the low/non-sensitization group withdrew from the study because of suffering from ankle trauma and one in the high-sensitization group was excluded because of deviating from the study protocol (received physical therapy for KOA at another hospital during the study period). The data from eight patients was missing at the early stage and therefore not included in the analysis. Completed data from patients randomized to the high-sensitization group (*n* = 14) was compared with those in the low/non-sensitization group (*n* = 14), see the flowchart in Fig. 2.

### Baseline characteristics

Of the 36 patients, 33 (91.7%) were females. Their median age was 56.5 years old (range 45 to 75; first and third quartiles 52.0–62.75), and median body mass index (BMI) was 22.9 kg/m^2^ (IQR 20.63–26.53). The disease duration ranged from 0.1 to 20 years (median = 3.5; first and third quartiles 0.25–7.00). Nearly 60% of the KOA patients were with bilateral involvement. The majority were diagnosed as Kellgren-Lawrence grade I (*n* = 17, 47.2%) or II (*n* = 13, 36.1%), and only 5 (13.9%) as grade III. All patients had previously received pharmaceutical treatment for KOA: traditional Chinese medicine (*n* = 7, 19.4%), calcium tablets (*n* = 12, 33.3%), glucosamine products (*n* = 8, 22.2%), or NSAIDs (*n* = 1, 2.8%). Only 8 (22.2%) had a history of acupuncture treatment and the median acupuncture treatment time was 7.5 times (first and third quartiles 2.5–10). At baseline, the median total WOMAC score was 49.80 (IQR 20.28–80.98). The median SF-12 physical health score was 37.44 (first and third quartiles 26.57–42.65), which was lower than the SF-12 physical mental score 50.29 (40.94–56.45) (Table 1).

**Table 1:** Baseline characteristics of participants

Variables	High-sensitization group (*n* = 18)	Low/non-sensitization group (*n* = 18)	*p*
Age, median (first and third quartiles), year	54.00 (51.50–60.25)	59.50 (51.50–66.25)	0.23^#^
Sex, *n* (%)			1.00^†^
Women	17 (94.4)	16 (88.9)	
Man	1 (5.6)	2 (11.1)	
Marital status, *n* (%)			1.00^†^
Married	18 (100.0)	17 (94.4)	
Single	0 (0.0)	1 (5.6)	
Height, median (first and third quartiles), cm	155.5 (152.00–160.75)	158.00 (155.00–160.00)	0.46^#^
Weight, median (first and third quartiles), kg	55.00 (52.75–60.25)	57.50 (51.50–66.00)	0.54^#^
BMI, median (first and third quartiles), kg/m^2^	23.30 (21.25–25.83)	23.14 (20.52–27.07)	0.67^#^
Education level, *n* (%)			0.41^†^
Illiteracy	1 (5.6)	2 (11.1)	
Primary school	1 (5.6)	1 (5.6)	
High school	5 (27.8)	10 (55.6)	
University	9 (50.0)	4 (22.2)	
Postgraduate	1 (5.6)	1 (5.6)	
Missing	1	0	
Duration of education, median (first and third quartiles), year	9.50 (8.00–12.5)	9.00 (6.00–12.00)	0.25^#^
Symptom duration, median (first and third quartiles), year	4.50 (1.25–12.00)	5.00 (1.00–8.50)	0.61^#^
Symptom duration, *n* (%)			1.00^†^
< 1 year	4 (22.2)	4 (22.2)	
1 to < 5 years	6 (33.3)	5 (27.8)	
5 to < 10 years	4 (22.2)	5 (27.8)	
≥ 10 years	4 (22.2)	4 (22.2)	
Target knees, *n* (%)			0.06^†^
Unilateral	5 (29.4)	11 (61.1)	
Bilateral	12 (70.6)	7 (38.9)	
Severe side of the bilateral knees			0.66^†^
Left	5 (41.7)	2 (28.6)	
Right	7 (58.3)	5 (71.4)	
Kellgren criteria, *n* (%)			0.43^†^
Kellgren 1	10 (58.8)	7 (41.2)	
Kellgren 2	6 (46.2)	7 (53.8)	
Kellgren 3	1 (20.0)	4 (80.0)	
Kellgren 4	0 (0)	0 (0)	
Missing	1		
Previous pharmaceutical treatment, *n* (%)			
Chinese medicine	3 (16.7)	4 (22.2)	1.00^†^
Calcium tablet	6 (33.3)	6 (33.3)	1.00^†^
Glucosamine products	4 (22.2)	4 (22.2)	1.00^†^
NSAIDs	1 (5.6)	0 (0)	1.00^†^
Others	0 (0)	2 (11.1)	0.49^†^
Previous acupuncture treatment, *n* (%)	1 (5.6)	7 (38.9)	0.04^†^
Frequency of acupuncture treatment, median (first and third quartiles)	10 (0)	5 (8)	0.57^#^
WOMAC Index, median (first and third quartiles)			
WOMAC total	49.85 (19.25–69.13)	49.05 (21.03–102.85)	0.84^#^
WOMAC pain	10.00 (5.50–21.68)	12.65 (6.60–19.98)	0.94^#^
WOMAC stiffness	2.40 (1.08–8.65)	5.30 (0.83–8.88)	0.58^#^
WOMAC function	31.00 (12.50–46.43)	31.30 (12.98–68.23)	0.82^#^
SF-12 Index, median (fi rst and third quartiles)			
Physical health	35.90 (27.11–42.05)	38.54 (25.91–44.44)	0.65^#^
Mental health	53.74 (47.45–56.68)	45.89 (37.66–56.20)	0.10^#^
Active range of motion, median (first and third quartiles)			
Flexion	115.50 (109.50–132.50)	112.00 (104.50–125.00)	0.14^#^
Extension	0 (0)	0 (0)	0.79^#^
Internal rotation	20.00 (15.75–30.25)	19.00 (12.75–26.00)	0.39^#^
External rotation	28.00 (24.50–30.25)	25.50 (20.00–31.50)	0.72^#^
Passive range of motion, median (first and third quartiles)			
Flexion	127.00 (121.75–145.00)	125.50 (110.25–135.00)	0.17^#^
Extension	0 (0)	0 (0)	NA
Internal rotation	29.00(24.25–35.00)	25.50 (22.00–31.75)	0.46^#^
External rotation	35.00 (28.25–36.50)	31.00 (24.00–37.25)	0.39^#^

Others including cervus and cucumis polypeptide injection and diacerein capsules

*NSAIDs* nonsteroidal anti-inflammatory drugs, *WOMAC* Western Ontario and McMaster Universities Osteoarthritis Index, *NA* not applicable

^#^Mann-Whitney *U* rank sum test

^†^Fisher’s exact test

### Outcomes

#### Primary outcome

Recruitment of 36 patients took 2 months, achieving the recruitment target. Retention rates were similar between the treatment groups, 14 (77.8%) patients in the high-sensitization group completed the 16-week follow-up and compared to 14 (77.8%) patients in the low/non-sensitization group. The mean number of treatment sessions attended (with 95% CI) was 11.2 (10.7 to 11.6) in the high-sensitization group and 11.4 (11.0 to 11.8) in the low/non-sensitization group. All patients received at least ten treatment sessions in total.

#### Secondary outcomes

**Fig. 3:**
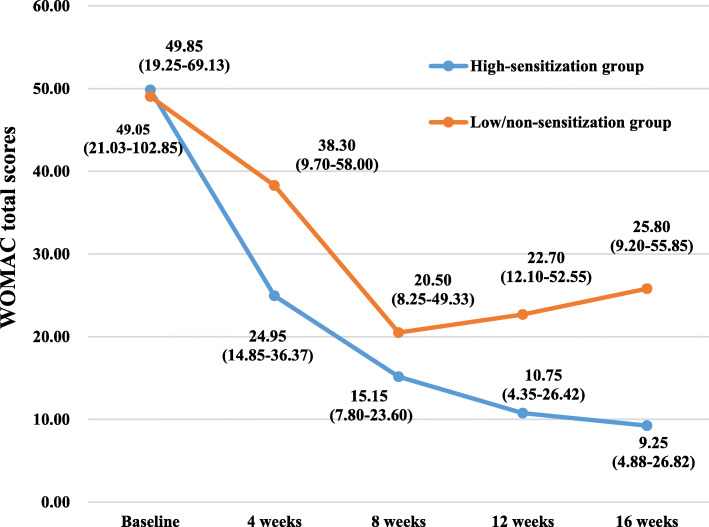
The effects of acupuncture on WOMAC total scores. The scores were shown as median (first and third quartiles)

**Fig. 4:**
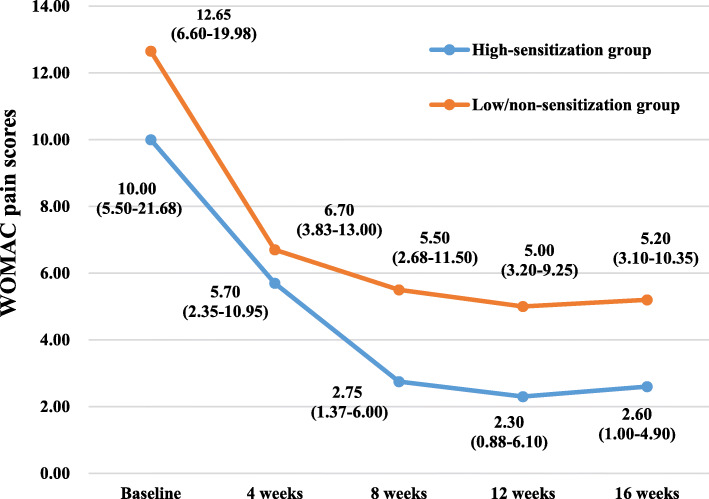
The effects of acupuncture on WOMAC pain scores. The scores were shown as median (first and third quartiles)

**Fig. 5:**
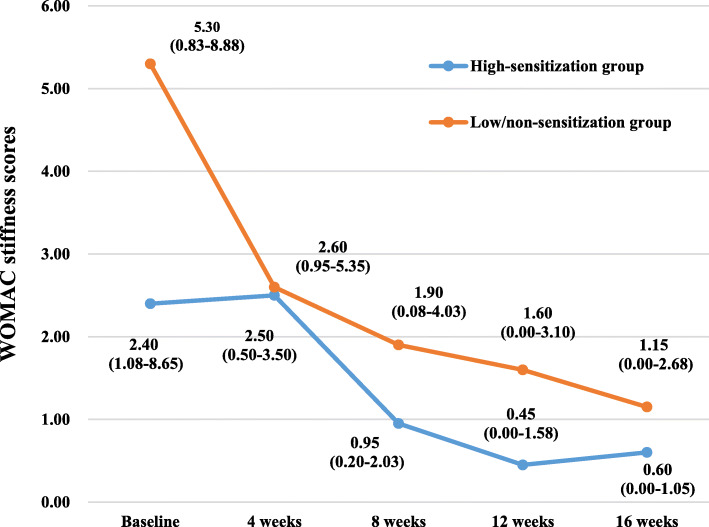
The effects of acupuncture on WOMAC stiffness scores. The scores were shown as median (first and third quartiles)

**Fig. 6:**
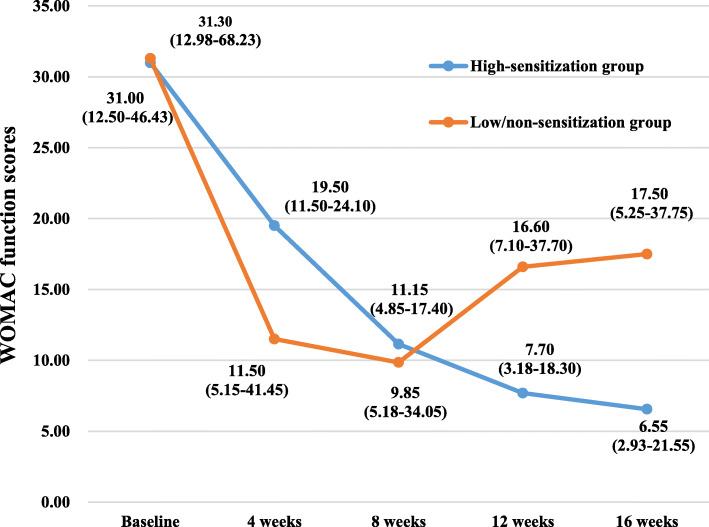
The effects of acupuncture on WOMAC function scores. The scores were shown as median (first and third quartiles)

**Fig. 7:**
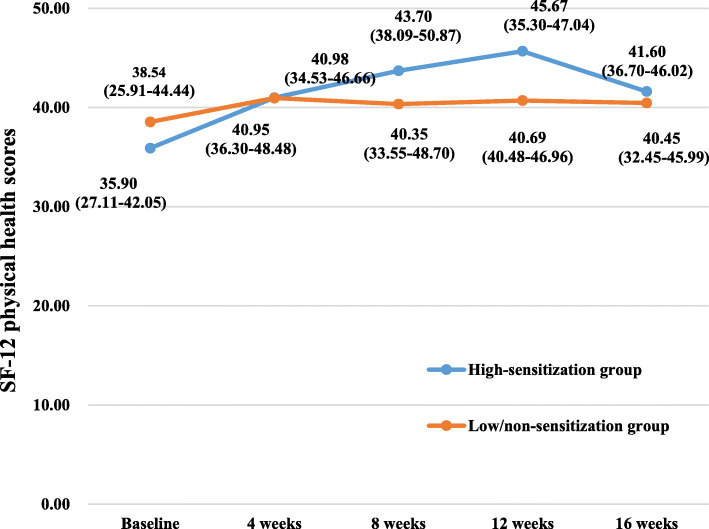
The effects of acupuncture on SF-12 physical health scores. The scores were shown as median (first and third quartiles)

**Fig. 8:**
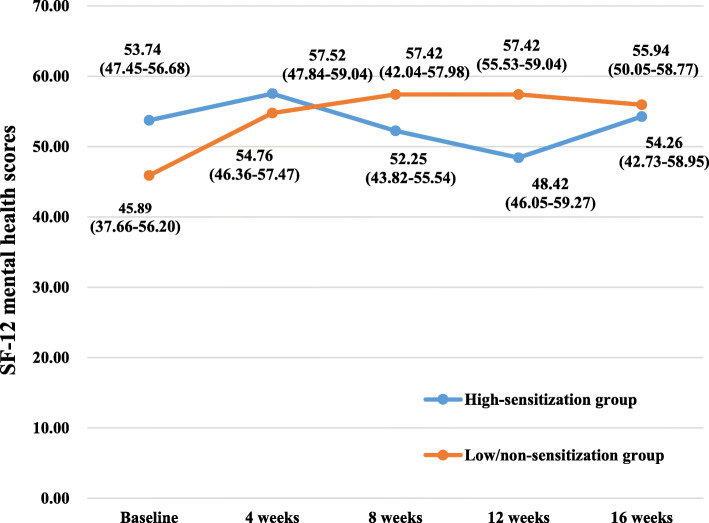
The effects of acupuncture on SF-12 mental health scores. The scores were shown as median (first and third quartiles)

The WOMAC scores and SF-12 scores of the participants in the two groups at various time points of the study are summarized in Table 2 and Figs. 3, 4, 5, 6, 7, and 8. The WOMAC total score decreased most from baseline to week 4, continued to decrease to week 8, then increased slightly in the low/non-sensitization group, while continuing to decrease in the high-sensitization group. Similar results were observed with the WOMAC subscales of pain, stiffness, and physical function. The WOMAC total score and the WOMAC subscales in the high-sensitization group were lower or very close to those in the control group at each assessment point. The analyses showed a significant change over time for WOMAC total score and subscales (*p* < 0.01), but the group-by-time interactions were not significant (*p* > 0.10). Also, repeated measures ANOVA did not show any between-group differences over time (*p* > 0.10).

**Table 2:** Effects of acupuncture on WOMAC and SF-12 over time, including comparison between groups over time

Groups	Times					Effect over time *P* value ^a^		
	Baseline (*n* = 36)	4 weeks (*n* = 35)	8 weeks (*n* = 28)	12 weeks (*n* = 28)	16 weeks (*n* = 28)	Group effect	Time effect	Group/time interaction
WOMAC index, median (first and third quartiles)								
WOMAC total								
High-sensitization group	49.85 (19.25–69.13) (95% CI 32.39–77.90)	24.95 (14.85–36.37) (95% CI 14.19–51.44)	15.15 (7.80–23.60) (95% CI 6.87–37.17)	10.75 (4.35–26.42) (95% CI 5.92–31.77)	9.25 (4.88–26.82) (95% CI 1.92–45.12)	0.38	< 0.001	0.67
Low/non-sensitization group	49.05 (21.03–102.85) (95% CI 39.55–97.44)	38.30 (9.70–58.00) (95% CI 19.39–61.78)	20.50 (8.25–49.33) (95% CI 13.40–43.87)	22.70 (12.10–52.55) (95% CI 15.07–49.78)	25.80 (9.20–55.85) (95% CI 12.03–49.75)			
WOMAC pain								
High-sensitization group	10.00 (5.50–21.68) (95% CI 8.11–19.47)	5.70 (2.35–10.95) (95% CI 3.19–11.79)	2.75 (1.37–6.00) (95% CI 1.61–8.76)	2.30 (0.88–6.10) (95% CI 1.15–7.97)	2.60 (1.00–4.90) (95% CI 0.54–9.67)	0.16	< 0.001	0.34
Low/non-sensitization group	12.65 (6.60–19.98) (95% CI 9.80–19.97)	6.70 (3.83–13.00) (95% CI 5.03–13.62)	5.50 (2.68–11.50) (95% CI 4.00–9.64)	5.00 (3.20–9.25) (95% CI 3.59–9.94)	5.20 (3.10–10.35) (95% CI 3.41–9.69)			
WOMAC stiffness								
High-sensitization group	2.40 (1.08–8.65) (95% CI 2.00–8.09)	2.50 (0.50–3.50) (95% CI 0.81–5.16)	0.95 (0.20–2.03) (95% CI 0.24–3.49)	0.45 (0.00–1.58) (95% CI 0.06–2.52)	0.60 (0.00–1.05) (95% CI − 0.40–4.01)	0.13	0.001	0.52
Low/non-sensitization group	5.30 (0.83–8.88) (95% CI 3.56–8.44)	2.60 (0.95–5.35) (95% CI 1.33–4.53)	1.90 (0.08–4.03) (95% CI 0.96–4.25)	1.60 (0.00–3.10) (95% CI 0.69–4.25)	1.15 (0.00–2.68) (95% CI 0.62–4.39)			
WOMAC function								
High-sensitization group	31.00 (12.50–46.43) (95% CI 20.50–52.12)	19.50 (11.50–24.10) (95% CI 9.61–35.08)	11.15 (4.85–17.40) (95% CI 4.78–25.16)	7.70 (3.18–18.30) (95% CI 4.47–21.54)	6.55 (2.93–21.55) (95% CI 1.60–31.62)	0.56	< 0.001	0.34
Low/non-sensitization group	31.30 (12.98–68.23) (95% CI 25.23–70.00)	11.50 (5.15–41.45) (95% CI 12.59–44.08)	9.85 (5.18–34.05) (95% CI 8.16–30.24)	16.60 (7.10–37.70) (95% CI 10.14–36.23)	17.50 (5.25–37.75) (95% CI 7.51–36.17)			
SF-12 index, median (first and third quartiles)								
Physical health								
High-sensitization group	35.90 (27.11–42.05) (95% CI 28.11–39.53)	40.98 (34.53–46.66) (95% CI 35.04–46.09)	43.70 (38.09–50.87) (95% CI 38.52–47.80)	45.67 (40.48–46.96) (95% CI 40.96–46.54)	41.60 (36.70–46.02) (95% CI 38.49–44.64)	0.56	< 0.001	0.81
Low/non-sensitization group	38.54 (25.91–44.44) (95% CI 31.55–45.76)	40.95 (36.30–48.48) (95% CI 40.60–47.34)	40.35 (33.55–48.70) (95% CI 36.97–46.10)	40.69 (35.30–47.04) (95% CI 37.79–46.58)	40.45 (32.45–45.99) (95% CI 35.24–45.47)			
Mental health								
High-sensitization group	53.74 (47.45–56.68) (95% CI 49.22–57.07)	57.52 (47.84–59.04) (95% CI 46.29–59.18)	52.25 (43.82–55.54) (95% CI 45.02–54.19)	48.42 (46.05–59.27) (95% CI 42.16–56.26)	54.26 (42.73–58.95) (95% CI 41.70–57.10)	0.32	< 0.001	0.25
Low/non-sensitization group	45.89 (37.66–56.20) (95% CI 37.90–53.79)	54.76 (46.36–57.47) (95% CI 46.74–55.31)	57.42 (42.04–57.98) (95% CI 43.59–55.43)	57.42 (55.53–59.04) (95% CI 51.97–59.18)	55.94 (50.05–58.77) (95% CI 49.26–57.63)			

^a^ repeated measurement data analysis of variance with square root transformation

SF-12 physical health scores increased from baseline to week 12, then decreased slightly at week 16 in the high-sensitization group; increased from baseline to week 4, then slightly decreased from week 8 to week 16 in the low/non-sensitization group. SF-12 mental health scores increased from baseline to week 4, then decreased from week 8 to week 12 in the high-sensitization group, while continued to increase in the low/non-sensitization group. Statistically significant changes over time were detected for SF-12 scores of both physical and mental health (*p* < 0.001). However, the group-by-time interactions were not significant (*p* > 0.10). No statistically significant differences were noted between groups over time (*p* > 0.10). In addition, no adverse events were reported for all the participants.

## Discussion

The aim of the present study was to assess the feasibility of a large trial of acupuncture for KOA with sensitized points. We confirmed the period to recruit 36 patients and demonstrated acceptable retention rates for participants in each treatment group and good patient adherence. Secondary outcomes suggested that acupuncture on the highly sensitized points may help to achieve improved therapeutic effects. This pilot trial has shown that a future full RCT is feasible.

Recruitment of 36 patients took 2 months in a single center, suggesting the possibility of conducting a multi-center clinical trial. Successful pilot targets were defined as 80% retention rate, but our result was slightly lower than expected. In our study, retention rates were similar between the treatment groups, 14 (77.8%) patients in the high-sensitization group completed the 16-week follow-up and compared to 14 (77.8%) patients in the low/non-sensitization group. One reason for patients lost to follow-up in our trial was that some patients did not fully understand the purpose of the study. Thus, we will vigorously publicize and explain the purpose and significance of the study through the Internet, WeChat, posters, and other channels to maximize patient participation in the main trial; meanwhile, we try to recruit patients around the trial center, regardless of patients who are far away. Another reason may be the fact that some patients may not have benefited from the acupuncture treatment. Therefore, one way to improve the retention rates is to distribute medication packages (without treatment effect) to all the included KOA patients and the KOA patients will be asked to come back to the hospital and change the package every 4 weeks during the follow-up period. Although we are confident that the medication package would help to achieve a higher follow-up rate, attention needs to be given to the follow-up procedures in a main trial.

Patients in the high-sensitization and low/non-sensitization groups received the treatment in accordance with protocol (three treatment sessions per week for four consecutive weeks, 12 treatment sessions in total), and all patients received at least ten treatment sessions in total. It may be attributable to the fact that the possibility of improvement in KOA condition and patients could receive free physical and chemical examination and treatment during the treatment period.

### Potential effectiveness of acupuncture treatment on sensitized points

The study was to assess feasibility; therefore, we did not expect to find statistical differences in WOMAC and SF-12 score between groups. In the PP analysis, there was a trend that the reduction of the total WOMAC score was greater in the high-sensitization group compared with the low/non-sensitization group, and the trend was most pronounced in the subscale of physical function. A relevant study has demonstrated that a 2.9 absolute change in the mean WOMAC function score indicates clinically significant improvement for acupuncture therapy of KOA [37]. In our trial, the decrease of the median physical functional score from baseline to 16 weeks post-randomization in the high-sensitization group was 24.45, much higher than that in the low/non-sensitization group. These results preliminarily suggested that acupuncture on the highly sensitized points may help to achieve improved therapeutic effects. However, as a pilot trial, its sample size was not powered to reach statistical significance for the outcomes examined. The results of the present trial should therefore be viewed as exploratory. The effectiveness of acupuncture treatment on sensitized points will be assessed in the main trial. To suit the relevance of repeated measurements by the same participant and the nesting of observations within study centers, we will use a generalized linear model with random effects (participants and research sites) to assess the intervention effect on outcome variables over time in the definitive trial.

### A waiting-list group

Lacking a blank control group, the pilot trial is not capable of determining whether clinical improvements are the consequence of treatment or spontaneous changes in the disease. Therefore, we will include a waiting-list group in the main trial. Patients in the waiting-list group will receive standard acupuncture treatment at the following acupoints after the trial is concluded: DUBI (ST35), NEIXIYAN (EX-LE4), YANGLINGQUAN (GB34), ZUSANLI (ST36), and XUEHAI (SP10). Several measures will be taken to maximize the patients’ retention. Non-acupuncture treatments, such as the application of medicinal liquor on the knee, heat therapy using the Teding Diancibo Pu (TDP) device, massage, and moxibustion, are allowed if patients in the waiting-list group request treatment during the study period.

### Strengths and limitations

The strengths of this pilot trial were that all the procedures were rigorously tested, including identification of eligible participants, recruitment, randomization, intervention delivery, and follow-up. As this is a pilot study, we cannot make any definitive conclusion regarding the effectiveness of acupuncture treatment of KOA on sensitized points. Also, this study has a few limitations. Firstly, our study was a single-center trial including 36 patients, there may be selection bias. However, in the main trial, we will recruit patients from multiple centers. Secondly, since most participants were lost to follow-up at the early stage for unknown reasons, the analyses were performed on the per-protocol (PP) population, intention-to treatment analysis was not used. Thirdly, lacking a blank control group, our study is not capable of determining whether clinical improvements are the consequence of treatment or spontaneous changes in the disease. Corresponding measures have been developed and will be taken in the main trial. Fourthly, there was an imbalance in the baseline history of acupuncture treatment, and this imbalance was not further analyzed due to the small sample size. Subgroup analyses by some baseline variables (such as BMI, severity of pain measured by VAS, and type of KOA) related to KOA will be conducted in the main trial. Fifthly, according to the study protocol, AEs were collected through case report forms at 4 weeks after the acupuncture treatment and followed at 8, 12, and 16 weeks, there may be recall bias. However, during the four consecutive weeks of acupuncture treatment, we recorded AEs in every treatment session (12 sessions in total) to aid recall. Finally, due to the nature of the intervention, it was not possible to blind acupuncturists to the group allocation. However, patients, outcome assessors, and data analysts were masked to minimize bias.

## Conclusions

Our pilot study has provided preliminary data on the feasibility of conducting a large trial to test the effectiveness of acupuncture at sensitized points in KOA patients. The results have revealed important limitations and options for improvement. These have enabled us to refine and modify the design of the main trial, such as including a waiting-list group and further measures to reduce attrition.

## Data Availability

The datasets generated during the current study are available from the corresponding author on a reasonable request.

## Abbreviations

ANOVA: Analysis of variance
IQR: Interquartile range
KOA: Knee osteoarthritis
SF-12: Short Form 12
VAS: Visual analog scale
WOMAC: Western Ontario and McMaster Universities Osteoarthritis Index

## References

[1] Manheimer E, Linde K, Lao L, Bouter LM, Berman BM (2007). Meta-analysis: acupuncture for osteoarthritis of the knee. Ann Intern Med.

[2] Itoh K, Hirota S, Katsumi Y, Ochi H, Kitakoji H (2008). Trigger point acupuncture for treatment of knee osteoarthritis--a preliminary RCT for a pragmatic trial. Acupunct Med.

[3] Manyanga T, Froese M, Zarychanski R (2014). Pain management with acupuncture in osteoarthritis: a systematic review and meta-analysis. BMC Complement Altern Med.

[4] Vas J, Mendez C, Perea-Milla E (2004). Acupuncture as a complementary therapy to the pharmacological treatment of osteoarthritis of the knee: randomised controlled trial. BMJ..

[5] McGettigan P, Henry D (2006). Cardiovascular risk and inhibition of cyclooxygenase: a systematic review of the observational studies of selective and nonselective inhibitors of cyclooxygenase 2. JAMA..

[6] Blower AL, Brooks A, Fenn GC (1997). Emergency admissions for upper gastrointestinal disease and their relation to NSAID use. Aliment Pharmacol Ther.

[7] Soni A, Joshi A, Mudge N, Wyatt M, Williamson L (2012). Supervised exercise plus acupuncture for moderate to severe knee osteoarthritis: a small randomised controlled trial. Acupunct Med.

[8] Hinman RS, McCrory P, Pirotta M (2014). Acupuncture for chronic knee pain: a randomized clinical trial. JAMA..

[9] Foster NE, Thomas E, Barlas P (2007). Acupuncture as an adjunct to exercise based physiotherapy for osteoarthritis of the knee: randomised controlled trial. Bmj..

[10] Jevsevar DS (2013). Treatment of osteoarthritis of the knee: evidence-based guideline, 2nd edition. J Am Acad Orthop Surg.

[11] McAlindon TE, Bannuru RR, Sullivan MC (2014). OARSI guidelines for the non-surgical management of knee osteoarthritis. Osteoarthr Cartil.

[12] Derry CJ, Derry S, McQuay HJ, Moore RA (2006). Systematic review of systematic reviews of acupuncture published 1996-2005. Clin Med (Lond).

[13] White AR, Filshie J, Cummings TM (2001). Clinical trials of acupuncture: consensus recommendations for optimal treatment, sham controls and blinding. Complement Ther Med.

[14] Ezzo J, Hadhazy V, Birch S (2001). Acupuncture for osteoarthritis of the knee: a systematic review. Arthritis Rheum.

[15] Kim EJ, Lim CY, Lee EY, Lee SD, Kim KS (2013). Comparing the effects of individualized, standard, sham and no acupuncture in the treatment of knee osteoarthritis: a multicenter randomized controlled trial. Trials..

[16] Chen R, Chen M, Xiong J, Yi F, Chi Z, Zhang B (2010). Comparison of heat-sensitive moxibustion versus fluticasone/salmeterol (seretide) combination in the treatment of chronic persistent asthma: design of a multicenter randomized controlled trial. Trials..

[17] Chen R, Chen M, Kang M (2010). The design and protocol of heat-sensitive moxibustion for knee osteoarthritis: a multicenter randomized controlled trial on the rules of selecting moxibustion location. BMC Complement Altern Med.

[18] Zhu B (2015). The plasticity of acupoint. Zhongguo Zhen Jiu.

[19] Chen M, Chen R, Xiong J, Yi F, Chi Z, Zhang B (2011). Effectiveness of heat-sensitive moxibustion in the treatment of lumbar disc herniation: study protocol for a randomized controlled trial. Trials..

[20] Li L, Fang J, Liu Z, Deng P, Lai X (2011). Clinical observation of thermal moxibustion in treating 50 cases of angina pectoris. Journal of New Chinese Medicine.

[21] Wei X, Xin S (2005). Study on pathogenesis of stroke by using infrared thermogram of the human back. J Tradit Chin Med.

[22] Chen R, Kang M (2006). A new type of disease response point - thermal point and its clinical significance. Journal of Jiangxi University of Traditional Chinese Medicine.

[23] Shen X, Wei J, Zhang Y (2006). Study on Volt-ampere (V-A) characteristics of human acupoints. Chinese Acupuncture & Moxibustion.

[24] Ben H, Li L, Rong PJ (2012). Observation of pain-sensitive points along the meridians in patients with gastric ulcer or gastritis. Evid Based Complement Alternat Med.

[25] Xie H, Chen R, Xu F, Song Y, Tang X, Li L (2012). Comparative study of heat-sensitive moxibustion in the treatment of knee osteoarthritis. Chinese Acupuncture & Moxibustion.

[26] Duan Q, Yuan F, Liang M, Dong J, Zhong P, Gu Z (2014). Clinical observation on thermal therapy of 120 cases of knee joint osteoarthritis treated by heat-sensitive moxibustion. Journal of New Chinese Medicine.

[27] Chen R, Kang M (2006). Acupoint heat-sensitization and its clinical significance. J Tradit Chin Med.

[28] XY Z. [The Guideline of the latest Chinese herbs to Clinical Research]2010. p. 478-479.

[29] Li H, Ma Z, Fang Z (2016). The rule of acupoints’ selection in acupuncture and moxibustion for knee osteoarthritis in clinic using data mining analysis. World Science and Technology/Modernization of Traditional Chinese Medicine and Materia Medica.

[30] Li D (2015). Literature research on acupoint selection rules and moxibustion methods in treatment of knee degenerative osteoarthritis by warming acupuncture. For all Health.

[31] Yuan HW, Ma LX, Zhang P (2013). An exploratory survey of deqi sensation from the views and experiences of chinese patients and acupuncturists. Evid Based Complement Alternat Med.

[32] Faik A, Benbouazza K, Amine B (2008). Translation and validation of Moroccan Western Ontario and McMaster Universities (WOMAC) osteoarthritis index in knee osteoarthritis. Rheumatol Int.

[33] Xie F, Li SC, Goeree R (2008). Validation of Chinese Western Ontario and McMaster Universities Osteoarthritis Index (WOMAC) in patients scheduled for total knee replacement. Qual Life Res.

[34] Cao XW, Guo D, Liu JW (2016). The efficacy and safety of the Shaoyao Shujin tablet for knee osteoarthritis: study protocol for a randomized controlled trial. Trials..

[35] Fang WH, Huang GS, Chang HF (2015). Gender differences between WOMAC index scores, health-related quality of life and physical performance in an elderly Taiwanese population with knee osteoarthritis. BMJ Open.

[36] Lam CL, Tse EY, Gandek B (2005). Is the standard SF-12 health survey valid and equivalent for a Chinese population?. Qual Life Res.

[37] Berman BM, Lao L, Langenberg P, Lee WL, Gilpin AM, Hochberg MC (2004). Effectiveness of acupuncture as adjunctive therapy in osteoarthritis of the knee: a randomized, controlled trial. Ann Intern Med.

